# Supplementary material for: Development of a Promising 18F-Radiotracer for PET Imaging Legumain Activity In Vivo
Source: Pharmaceuticals (Basel). 2022 Apr 27;15(5):543. doi: 10.3390/ph15050543 (PMC9145320; doi:10.3390/ph15050543)
Supplement: Supplementary file 1 [file pharmaceuticals-15-00543-s001.zip › pharmaceuticals-1608008-supplementary.pdf]

Development of a Promising ^{18}F-Radiotracer for PET Imaging Legumain Activity In Vivo

Chunmei Lu[1,2], Xiuting Wang[1,2], Qiqi Wang[1,2], Lixia Zhang[2] Jianguo Lin[2*] & Ling Qiu[1,2*]

1. School of Chemical and Material Engineering, Jiangnan University, Wuxi 214122, China.
2. NHC Key Laboratory of Nuclear Medicine, Jiangsu Key Laboratory of Molecular Nuclear Medicine, Jiangsu Institute of Nuclear Medicine, Wuxi 214063, China

Supplementary Figures

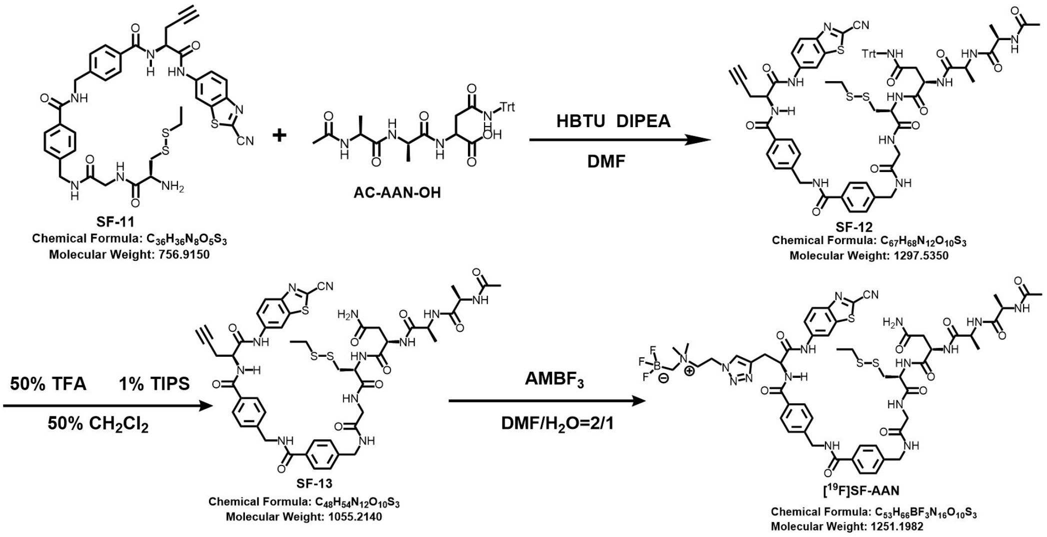

Figure S1. Synthetic route of nonradioactive probe [^{19}F]**SF-AAN**.

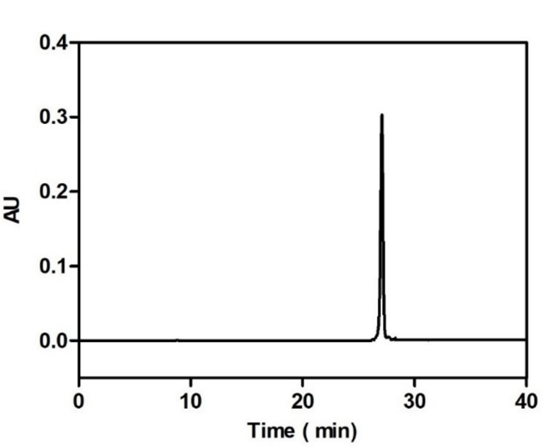

Figure S2. HPLC trace of compound **SF-12**.

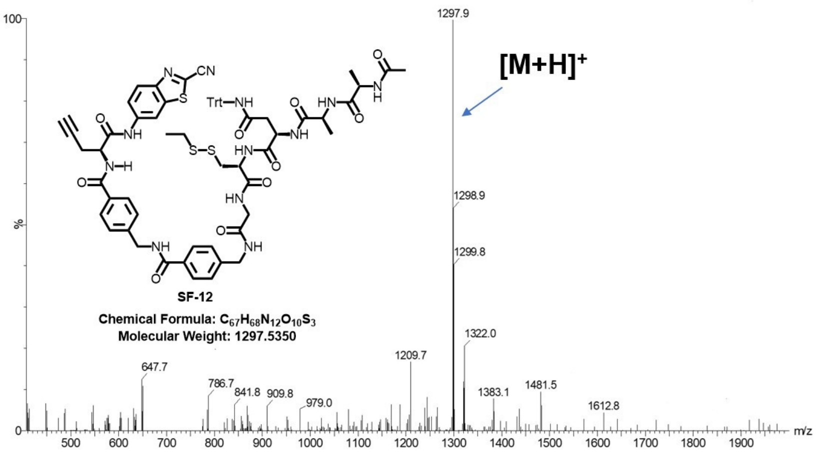

Figure S3. ESI-MS of compound **SF-12**.

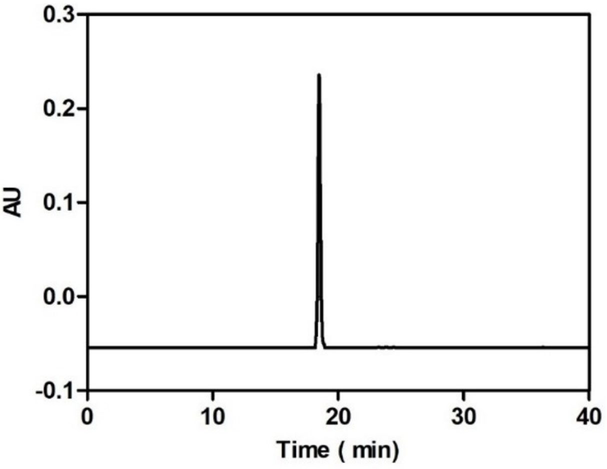

Figure S4. HPLC trace of compound **SF-13**.

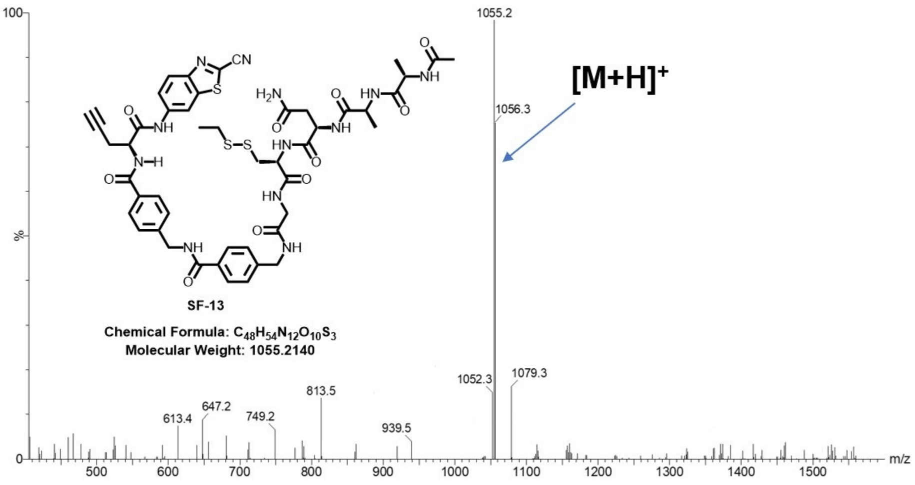

Figure S5. ESI-MS of compound **SF-13**.

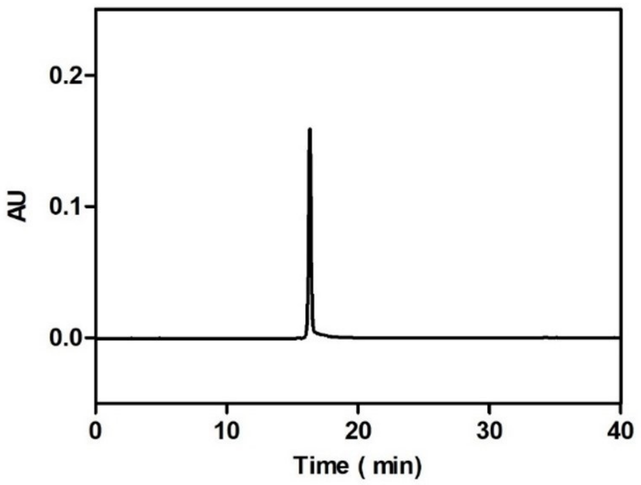

Figure S6. HPLC trace of nonradioactive probe [^{19}F]**SF-AAN**.

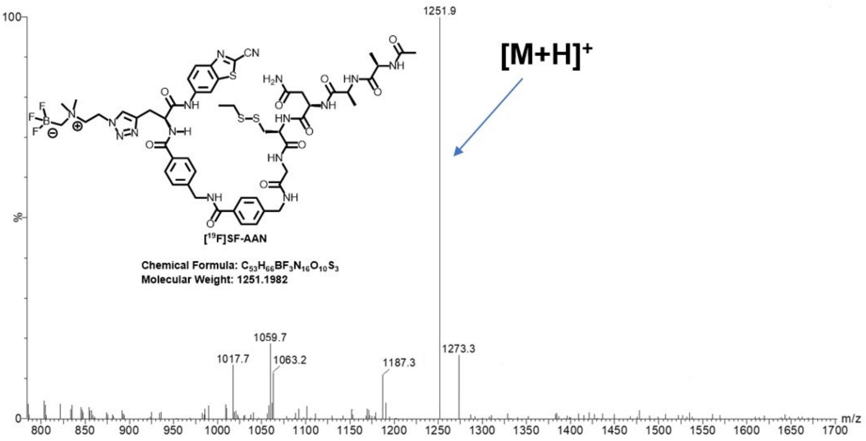

Figure S7. ESI-MS of nonradioactive probe [^{19}F]**SF-AAN**.

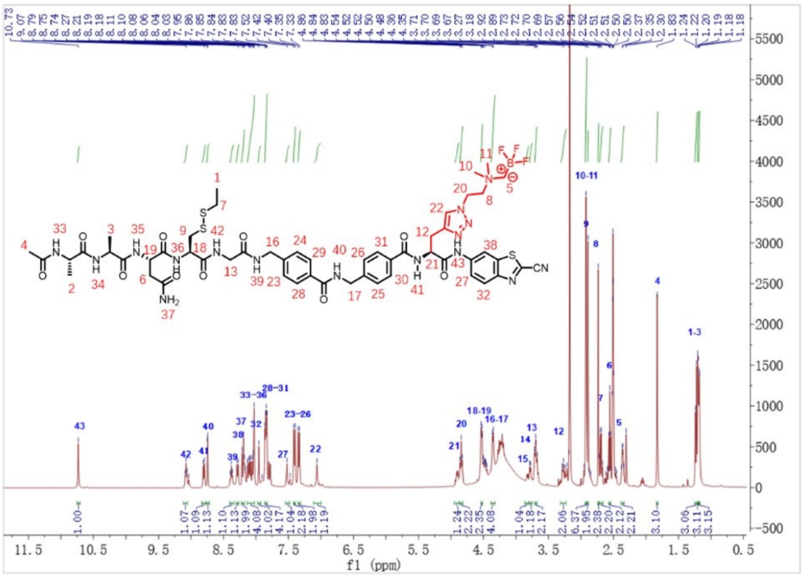

Figure S8. ^{1}H-NMR of nonradioactive probe [^{19}F]**SF-AAN**.

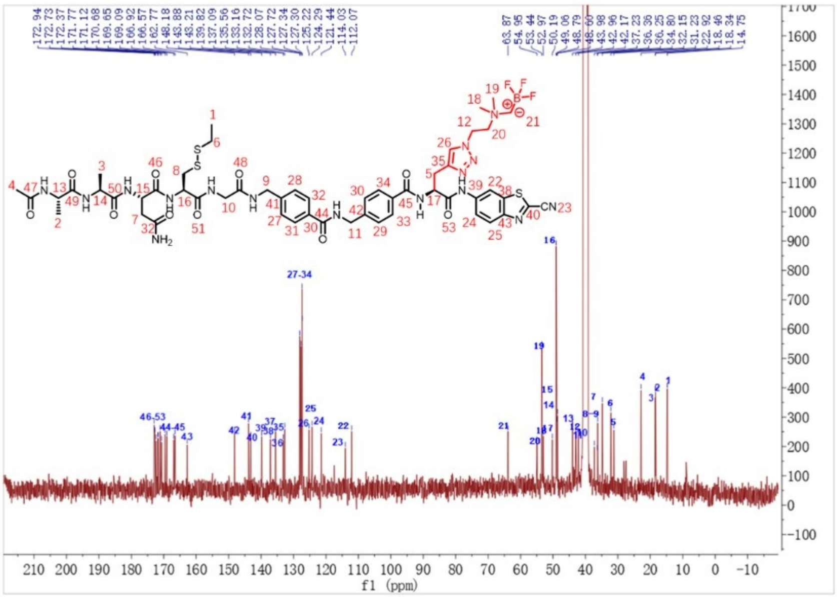

Figure S9. ^{13}C-NMR of nonradioactive probe [19F]**SF-AAN**.

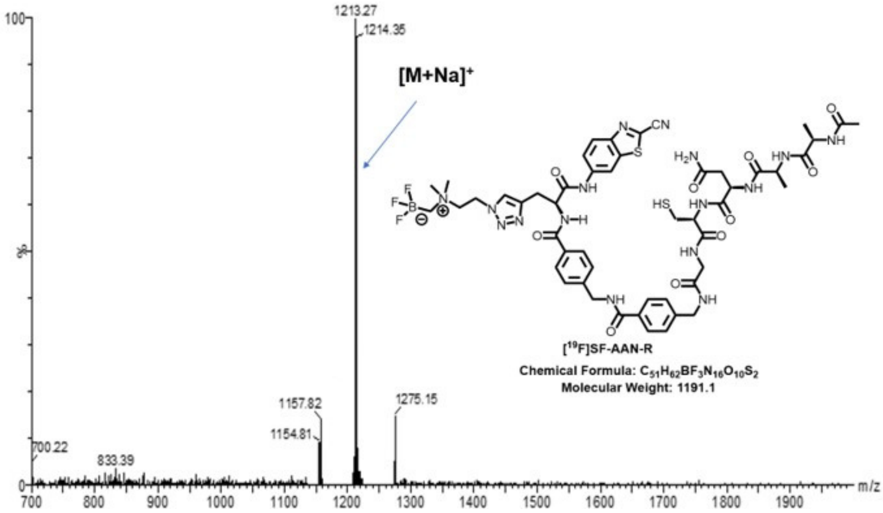

Figure S10. ESI-MS of compound [19F]**SF-AAN-R**.

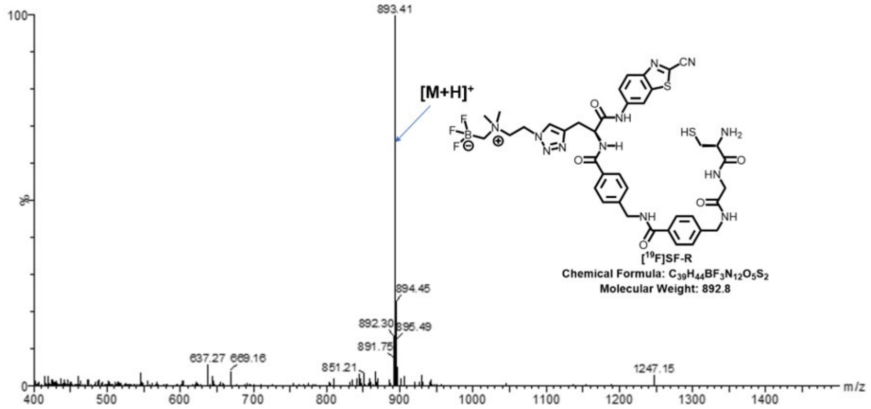

Figure S11. ESI-MS of compound [19F]**SF-R**.

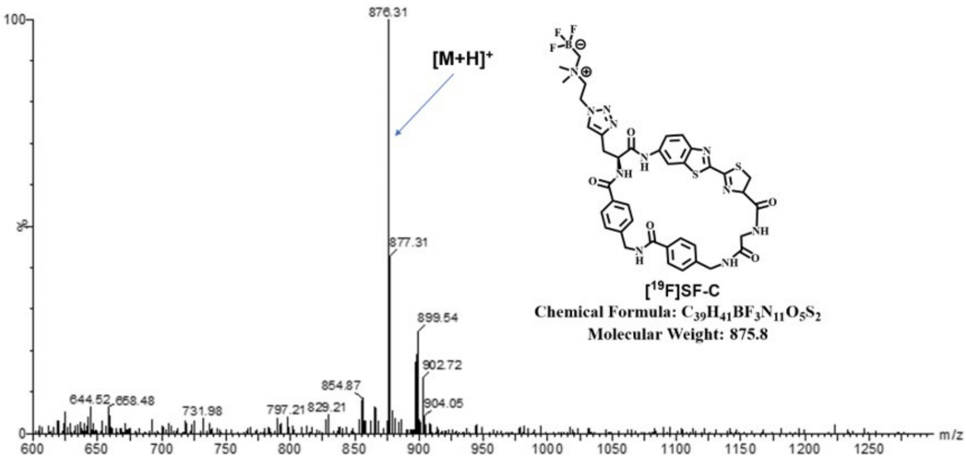

Figure S12. ESI-MS of compound [^{19}F]**SF-C**.

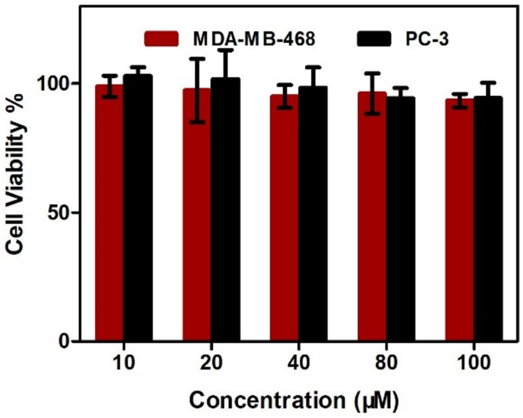

Figure S13. Cytotoxicity assay of nonradioactive probe [^{19}F]**SF-AAN** against MDA-MB-468 and PC-3 cells at 24 h.

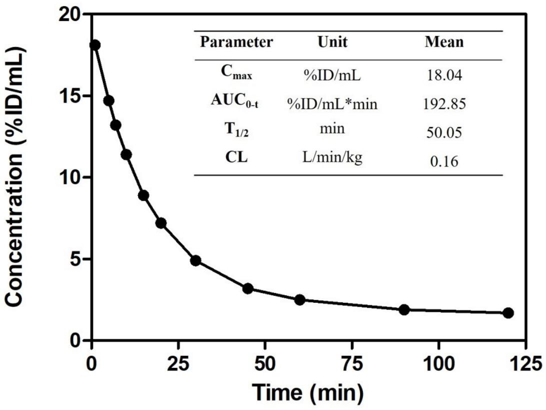

Figure S14. Pharmacokinetics of [^{18}F]**SF-AAN** in BALB/c normal mice within 2 h.

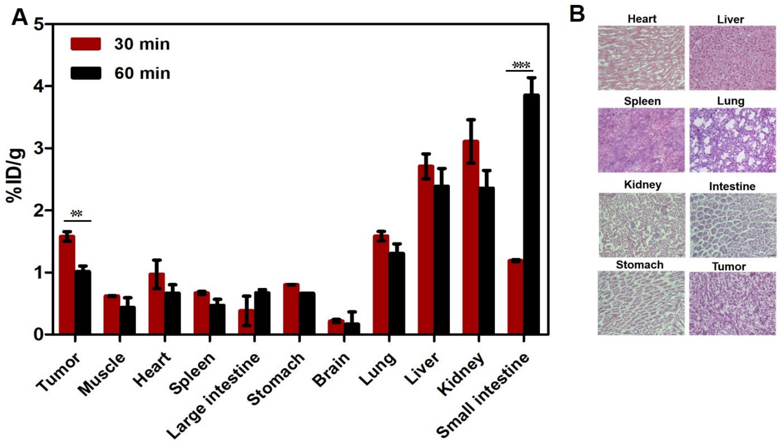

Figure S15. (A) Biodistribution of [^{18}F]**SF-AAN** in MDA-MB-468-bearing mice at 30 and 60 min post injection. Error bar represents standard deviation (n = 3). ** denotes p < 0.01; *** denotes p < 0.001. (B) H&E staining of vital organs and tumor of MDA-MB-468-bearing mouse. Scale bar: 50 μm.

Supplementary Tables

Table S1. HPLC conditions for analysis of all compounds (Method A).

Time/min	Flow (mL/min)	H_2O (0.1% TFA) %	MeCN (0.1% TFA) %
Initial	1	80	20
3	1	80	20
35	1	10	90
40	1	80	20

Table S2. HPLC conditions for purification of nonradioactive probe [^{19}F]**SF-AAN** (Method B).

Time/min	Flow (mL/min)	H_2O (0.1% TFA) %	MeCN (0.1% TFA) %
Initial	3	75	25
3	3	75	25
25	3	60	40
30	3	10	90
35	3	75	25
